# Multigenerational effects of uranium exposure reveal stronger testicular dysregulation in the second generation

**DOI:** 10.1016/j.crtox.2025.100279

**Published:** 2025-12-26

**Authors:** Audrey Legendre, Céline Gloaguen, Dimitri Kereselidze, Nawel Saci, Sophia Murat El Houdigui, Pascal Froment, Christelle Elie, Catherine Defoort, Philippe Lestaevel, Mohamed Amine Benadjaoud, Maâmar Souidi, Stéphane Grison

**Affiliations:** a Autorité de Sûreté Nucléaire et de Radioprotection (ASNR), PSE-SANTE/SESANE/LRTOX, PSE-SANTE/SERAMED/LRACC, USNR/SEARCH, F-92260 Fontenay-aux-Roses, France; b CNRS, IFCE, INRAE, University of Tours, PRC, F-37380 Nouzilly, France; c Centre Cardiovasculaire et Nutrition (C2VN), INRAE, INSERM, Aix Marseille Université, 13005 Marseille, France

**Keywords:** Uranium, Multigenerational exposure, Low-dose, Endocrine disruption, Testicular toxicity

## Abstract

**Background:**

Infertility is a significant public health issue that can be influenced by environmental pollutants. As a radioactive heavy metal and environmental contaminant, uranium has the potential to impact fertility.

**Objective:**

This study assesses the multigenerational reproductive effects of chronic, non-nephrotoxic uranium exposure across three generations of male rats.

**Methods:**

In this study, a non-nephrotoxic uranium solution (40 mg/L) was chronically administered *via* drinking water to male and female F0 rats (n = 20 per group) throughout their lifespan. The objective was to evaluate the potential reprotoxic effects of uranium on males across three generations (F0, F1, F2), with a focus on spermatogenesis, steroidogenesis, and testicular homeostasis, including oxidative stress, inflammation, apoptosis, and vitamin D metabolism.

**Results:**

Steroidogenesis was modulated in all generation, with dysregulation of sex and pituitary hormones (testosterone, estradiol, gonadotropins, Luteinizing Hormone (LH), Follicle-Stimulating Hormone (FSH). Morphological and histological changes in the testes were observed in both the F1 and F2 generations. Spermatogenesis was dysregulated by an increased proportion of seminiferous tubules at stage I-VI and reduced expression of *TH2B* and *eppin* mRNA. Interestingly, gene expression analysis of several markers involved in the regulation and protection of testicular homeostasis revealed significant effects only on the F2 generation. In this generation, uranium exposure also disrupted vitamin D metabolism in the testes.

**Conclusion:**

Uranium may impair testicular function, with more pronounced effects observed in the F2 generation. These findings highlight its potential for multigenerational toxicity and underscore the need for further research into its impact on human reproductive health.

## Highlights

• Exposure to a non-nephrotoxic dose of uranium impacts testicular function in rats.
• Multigenerational exposure (F0–F2) in rats used to assess reproductive effects.
• Major dysregulations observed in F2 rats.
• Clarified effects on spermatogenesis, steroidogenesis and testicular homeostasis.
• Findings contribute to risk assessment and radiation protection.

## Graphical abstract

**Figure:**
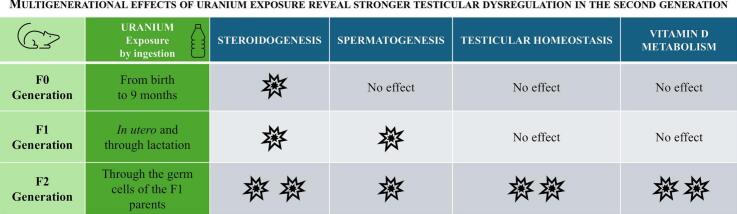

## Introduction

Infertility is a global public health issue with multifactorial origins (Cox et al., 2022); affecting both men and women. The percent of infertility that is attributable to males ranged between 20–70 % depending of considered areas / the world area (Agarwal et al., 2015). In men, half of infertility cases are linked to defects in spermatogenesis, while the rest are considered idiopathic. Male infertility may involve morphological or endocrine-related disruptions in spermatogenesis.

Endocrine disruptors are increasingly recognized for their negative impact on reproductive health (Lahimer et al., 2023). Many idiopathic cases lack identifiable physiological causes (Jodar et al., 2015, Raperport et al., 2024); with environmental pollutants and deteriorating living conditions among the leading hypotheses (Oliva et al., 2001, , xxxx). Anthropogenic pollution, including lifestyle factors such as alcohol consumption, smoking, high-fat diets, and stress, contributes to oxidative stress and endocrine disruption. According to the World Health Organization (WHO) (World Health Organization, 2023); environmental degradation is associated with declining fertility in exposed individuals and their descendants. Exposure during critical windows, such as gestation or the preconception period, can interfere with development and lead to intergenerational effects (Grison et al., 2024).

Among environmental pollutants, uranium is particularly concerning because of its toxic effects on reproductive health. Uranium is a naturally occurring radioactive metal (Wu et al., 2014); that enters the environment through erosion or industrial and military activities, posing risks via contaminated water or airborne dust. Its health effects stem from both radiological and chemical properties, although natural and depleted uranium are primarily chemotoxic (Craft et al., 2004, Souidi et al., 2009). Uranium exposure can impair fertility by affecting multiple reproductive organs (Wang et al., 2020).

Experimental studies have linked uranium dust exposure to reduced fertility in rats through several mechanisms, including hormonal disruption, altered gene expression, protein damage, and inflammation caused by oxidative stress (Legendre et al., 2019, Legendre et al., 2016, Toklayeva et al., 2019). Testicular injury and decreased testosterone levels have been associated with the ROS–hnRNP A2/B1–COX-2 signaling pathway (Lu et al., 2021). Uranium also induces epigenetic modifications in gonadal tissue (Elmhiri et al., 2018) and endocrine disturbances (Grison et al., 2022). Furthermore, it disrupts vitamin D metabolism, affecting the vitamin D receptor (VDR), its target genes, and the retinoid X receptor alpha (RXRα) (Tissandie et al., 2008, Tissandie et al., 2007). Since vitamin D plays a key role in regulating sperm motility and calcium homeostasis in the testes, its deficiency may contribute to infertility. Meta-analyses support the use of vitamin D supplementation to improve sperm motility (Arab et al., 2019, Tania et al., 2023, Yahyavi et al., 2024).

Beyond individual effects, uranium exposure can induce heritable reproductive alterations. Chronic low-dose exposure in rats has been shown to cause sperm abnormalities and reduced fertility in F1 offspring, despite normal sperm counts. In the F2 generation, global DNA hypomethylation in sperm was observed (Legendre et al., 2019); along with sex-specific methylation imbalances in gonadal tissue across both F1 and F2 generations, and altered expression of genes involved in DNA methylation (Elmhiri et al., 2018). These epigenetic modifications may affect both fertility and transgenerational inheritance, beginning with the next generation (F3). Additionally, metabolomic profiling revealed persistent alterations in sperm composition across generations (Grison et al., 2022).

Building on previous analyses conducted under comparable multigenerational animal exposure protocols aimed at evaluating the risk of adverse reproductive effects in subsequent generations, which included morphometric assessments, global DNA methylation profiling, and sperm metabolomic characterization (Legendre et al., 2019, Grison et al., 2022); this study extends beyond the previously reported disruption of testicular global methylation (Elmhiri et al., 2018). It investigates male reproductive function by examining spermatogenesis, steroidogenesis, and testicular homeostasis across three successive generations. The objective is to provide an integrated perspective on the multigenerational impact of chronic uranium exposure on rat reproductive health.

## Material & methods

### Radionuclide concentration

Natural uranium (NU) was obtained from CERCA (Pierrelatte, France) (Grison et al., 2013). NU (Olympic Dam) was dissolved in mineral water and prepared in its uranyl nitrate hexahydrate (UO_2_(NO_3_)_2_, 6H_2_O) to a final uranium concentration of 40 mg.L^-1^. The specific activity of NU is 2.42 × 10^+4^ Bq⋅g^−1^ and its isotopic composition is ^238^U: 99.307 %, ^235^U: 0.688 % and ^234^U: 0.005 %. The final concentration of natural uranium (NU) in drinking water was set at 40 mg/L, approximately three times higher than the maximum uranium level of 12.4 mg/L naturally found in Finnish well water (Salonen, 1994). This corresponds to an average daily intake of about 1 mg of uranium per rat. Given that intestinal absorption of uranyl in rats is estimated at only 0.06 %; roughly 0.6 µg of uranium would enter the bloodstream. As previously demonstrated by the lack of significant differences in blood counts and biochemical markers, including plasma and urine proteins, ions, and liver, cardiovascular, renal indicators as well as creatinine clearance, this non-nephrotoxic daily intake dose of NU (Grison et al., 2013) can be considered low, despite its relatively high environmental levels. The kidneys are the primary organs affected by uranium toxicity.

Radioactive waste, including biological materials, effluents, and contaminated bedding, was collected and disposed of by ANDRA (Châtenay-Malabry, France), an authorized facility specializing in radioactive waste management.

## Animal models and experimental Design

### Animal housing

Twelve-week-old Sprague-Dawley rats, 16 days pregnant, were obtained from Charles River Laboratories (L’Arbresle, France). They were housed individually under controlled conditions: a 12-hour light/12-hour dark cycle, temperature maintained at 21 °C, and relative humidity at 50 %. Animals had, *ad libitum* access to a standard rodent pellet diet and water. Male offspring were housed in pairs, each with a pup from a different dam, randomly assigned.

All experimental procedures were approved by the Animal Ethics Committee of the Institute of Radioprotection and Nuclear Safety (now Nucléaire et de Radioprotection (ASNR)) and complied with French regulations on animal experimentation (European Directive 2010/63/UE and Ministry of Agriculture Act No. 2013-118, February 1, 2013) and were conducted within the PARRAD platform (agreement E92-032-01).

## Experimental design

Male and female F0 Sprague-Dawley rats were chronically exposed to uranyl nitrate hexahydrate (NU) from birth through ad libitum consumption of drinking water containing 40  mg·L^−1^ NU for the entire 9-month protocol. This concentration corresponds to an estimated daily intake of approximately 1  mg per rat. Exposure continued until the animals reached 9 months of age, simulating chronic environmental exposure in human populations. Control dams received uncontaminated drinking water throughout the same period.

At six months of age, both exposed and unexposed male and female F0 rats were mated to produce the F1 generation, allowing assessment of the effects of parental uranium exposure on their offspring.

F1 male and female offspring were exposed to NU exclusively through maternal milk during lactation, up to weaning on postnatal day 21 (Wappelhorst et al., 2002). At six months of age, both exposed and unexposed F1 rats were mated to generate the F2 generation. The F2 offspring, comprising both males and females, were indirectly exposed to NU solely via the germline of the F1 generation.

## Sampling and zoometric data analysis

**Fig. 1:**
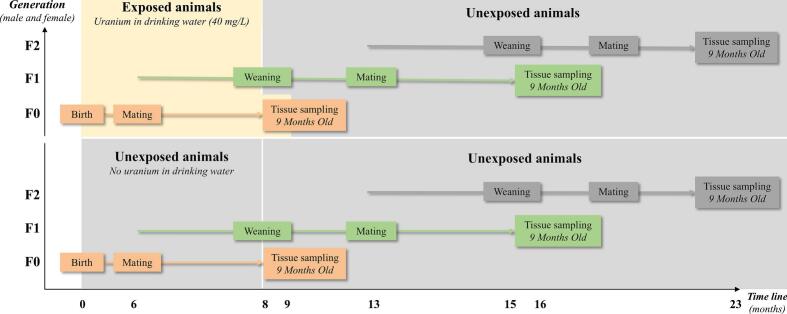
Experimental multigenerational protocol. Three generations of male and female rats (F0, F1, and F2; n = 20) were monitored. F0 animals (orange) were exposed to natural uranium (NU) via drinking water from birth for a duration of 9 months, while control animals received uncontaminated water. The F1 generation (green) was exposed in utero and through lactation via their F0 mothers until weaning. After weaning, all groups, including F1, received uncontaminated water ad libitum. The F2 generation (grey) was exposed to uranium only indirectly, through the germ cells of their F1 parents.

In each generation, rats were euthanized at 9 months of age following deep anesthesia induced by 5 % isoflurane inhalation (Abbott France, Rungis, France), followed by intracardiac puncture. Reproductive organs, including testes, epididymides, and seminal vesicles, were collected and weighed. One testis was snap-frozen in liquid nitrogen and stored at − 80 °C, while the contralateral testis was processed for histological analysis (Fig. 1).

Since reproductive organ weight data consists of paired measurements within the same animal, it is reasonable to assume that these observations are correlated. A linear mixed-effects model was applied using the lme4 package in R to account for within-subject correlation and to avoid underestimating standard errors or increasing the risk of false positives (version 1.1–37).

## Histological analysis of testicular tissue

Following removal, the testes were fixed in Bouin’s solution for 24 h and subsequently decapsulated. The tissues were dehydrated, embedded in paraffin, and sectioned at a thickness of 5  µm. Sections were mounted on SuperFrost glass slides (Labonord, Templemars, France) for histological staining. Deparaffinization was followed by staining with periodic acid–Schiff (PAS) reagent and counterstaining with hemalum (PAS-H). Slides were mounted using Eukitt mounting medium (O. Kindler GmbH, VWR).

Images were captured using a Leica DM 4000B microscope (Microvision, Évry, France) equipped with a SONY XCD-U100CR digital camera. Histological analysis of seminiferous tubules (ST) was performed using Histolab software (Microvision Instruments, Évry, France).

Morphological evaluation included measurements of ST diameter and area, as well as identification of abnormalities such as vacuolization, Sertoli cell-only (SCO) tubules, and multinucleated giant cell formation. Germ cell types were identified, and tubules were staged according to the morphological classification system of Clermont and Perey et al. (Clermont and Perey, 1957).

Following the approach described in (Legendre et al., 2016, Colpi et al., 2021), A total of 100 cross-sections of seminiferous tubules were analyzed per animal, with sample sizes ranging from 5 to 7 animals per group for both the F1 and F2 generations.

• Seminiferous tubule vacuolization was defined as the presence of one or more vacuoles of greatest diameter ≥ 16 μm located within 1 cell layer of the ST basement membrane.
• When a major: minor axis was less 1.5:1, tubules were counted and only the minor diameter axis was averaged for each animal and each group.
• The volume of Leydig cells was evaluated following this formula:
  $$\mathrm{Volume}=\frac{\mathrm{diameter}\times \pi }{6}$$
  Fifty cells were measured per animal.
• Staging was performed and analyzed as % of ST in stage I-VI, stage VII-VIII, stage XIV.
• The mean of the number of SC nuclei per ST in stage VII-VIII (n = 10 ST per animal) was evaluated.

The data were presented as the mean ± SD. A linear mixed effect model was employed using the lme4 Package in R (1.1–37) for histomorphometry analysis, diameter and lumen of ST, and Leydig cell measurement. A Poisson or quasi-Poisson regression was conducted for the anomaly rates modeling (vacuoles, GCL, TSL, and staging) using the GLM function from R software (R version 4.4.0).

## Gene expression levels

Quantitative reverse transcription PCR (RT-qPCR) was performed on total RNA extracted from testicular tissue. Frozen testes (excluding the tunica albuginea) were thawed and homogenized in M199 medium (Sigma-Aldrich) using ten manual strokes with a Potter–Elvehjem homogenizer. Total RNA was isolated using the RNeasy Mini Kit (Qiagen, Courtaboeuf, France), following the manufacturer’s protocol. Prior to cDNA synthesis, RNA quality was evaluated by spectrophotometry (NanoDrop™ 2000, Thermo Fisher Scientific, France) to determine concentration and purity (A260/A280 and A260/A230 ratios). RNA integrity was then confirmed by chip-based electrophoresis using a Bioanalyzer 2100 (Agilent Technologies, France). Samples with an RNA Integrity Number (RIN) ≥ 7 were retained for downstream analysis, which was performed with 1  µg of total RNA using the High-Capacity cDNA Reverse Transcription Kit (Applied Biosystems, Courtaboeuf, France).

Real-time PCR was conducted using 10  ng of cDNA per reaction on a QuantStudio™ 7 Flex Real-Time PCR System (Applied Biosystems), with SYBR Green PCR Master Mix (Applied Biosystems). Gene expression was normalized using the geometric mean of three stably expressed reference genes in rat testis: β-actin (*βact*), β2-microglobulin (*βmg2*), and hypoxanthine–guanine phosphoribosyltransferase (*HPRT*), as recommended by Vandesompele et al., to minimize variability and enhance statistical precision (Vandesompele et al., 2002).

Relative quantification was calculated using the actual amplification efficiency for each primer pair. Spermatogenesis, steroidogenesis and testicular homeostasis were explored at the molecular level:

*Spermatogenesis function:*

• Germ cell markers: *c-kit R, TH2B, TP2, eppin*
• Sertoli cell markers: *eppin, transferrin*

*Steroidogenesis function:*

• Steroid hormone metabolism: *GATA4, GATA6, SF-1, Dax-1, StAR, Cyp11A1, Cyp17A1, 3β-HSD1, 17β-HSD3, Cyp19A1, 5α-R1, ApoD*
• Cholesterol metabolism: *Cyp27A1*
• Receptors: *FSHR, LRHR, AR, ERα*

*Testicular homeostasis*.

• Sertoli cell secretory products: *TGF-β1, TNF-α*
• Blood-testis barrier (BTB) integrity: *β-catenin, N-cadherin, claudin-11, occludin, connexin*
• Oxidative stress: *Cat, Cu-Zn SOD (SOD1), Mn-SOD (SOD2), GPX, GST, Nrf2*
• Inflammation: *IL-1β, IL-6, IL-8*
• Apoptosis: *Fas, FasL, BID, Bak, Bax, Bcl-2, caspase-3, caspase-9, annexin V*
• Vitamin D pathway: *VDR, RxRα, Cyp24A1, Cyp2R1, Cyp27B1*

(See Supplemental Table 1 for full gene list and primer details.)

All data were expressed as mean ± standard deviation (SD) and analyzed using SigmaPlot software (Systat Software Inc., San Jose, CA, USA). Data normality was assessed using the Shapiro–Wilk test. If the assumption of equal variance was met, parametric tests were applied; otherwise, the Mann–Whitney test was used. Statistical significance was set at p < 0.05.

## Hormonal measurements

Hormonal assays were conducted on both plasma and testicular extracts. Blood samples were collected using heparinized syringes, centrifuged at 950 × g for 5 min, and the resulting plasma supernatants were immediately stored at − 80 °C for later analysis. Plasma concentrations of testosterone, estradiol, luteinizing hormone (LH), follicle-stimulating hormone (FSH), and vitamin D metabolites, 25(OH)D, 1,25(OH)_2_D, and 24,25(OH)D, were quantified.

Testes were thawed and homogenized in cold phosphate-buffered saline (PBS) without Ca^2+^ and Mg^2+^ using an Ultra-Turrax T25 homogenizer (JANKE & KUNKEL, IKA® Labortechnik, Germany). Following ultracentrifugation at 20,625 × g for 15 min at 4 °C, protein concentrations were determined using the Bradford method (Bio-Rad Protein Assay, Bio-Rad, Marnes-la-Coquette, France). Testicular extracts were used to measure intratesticular testosterone and estradiol levels.

Plasma and testicular testosterone concentrations were assessed using a radioimmunoassay (RIA) with tritiated testosterone, as previously described (Hochereau-de Reviers et al., 1990); with a sensitivity of 15 pg/tube and an inter-assay coefficient of variation (CV) of 5.3 %. Estradiol (in both plasma and testicular extracts) and plasma FSH levels were determined using competitive inhibition enzyme immunoassays (estradiol: CEA461Ra; FSH: CEA830Ra; Euromedex, Souffelweyersheim, France), following the manufacturer’s protocols. The minimum detectable concentrations were 4.75 pg/mL for estradiol and 0.92 ng/mL for FSH, with intra-assay CVs below 10 % and no cross-reactivity with analogues. Intratesticular concentrations were expressed as ng/gram of testis.

Plasma LH concentrations were measured by radioimmunoassay (RIA) using reagents from the National Institute of Diabetes and Digestive and Kidney Diseases (NIDDK), including rat LH-RP-3 for standard calibration and rabbit polyclonal antiserum, as previously described (Froment et al., 2002). The assay sensitivity was 20 pg/tube, with an inter-assay CV of 18 %. All samples were analyzed in duplicate.

Vitamin D metabolites in plasma, 25(OH)D, 1,25(OH) _2_D, and 24,25(OH)D, were quantified using liquid chromatography–tandem mass spectrometry (LC–MS/MS), following protocols described by Urakami et al. (2000) and sample preparation methods from Wang et al. (Urakami et al., 1999, Urakami et al., 2000). Results were expressed as mean ± standard deviation (SD).

Statistical analyses were performed using SigmaPlot software (Systat Software Inc., San Jose, CA, USA). Data normality was assessed using the Shapiro–Wilk test. If variance was homogeneous, an equal variance test was applied; otherwise, the Mann–Whitney test was used. For vitamin D data, one-way ANOVA followed by the Tukey–Kramer post hoc test and two-way ANOVA were used to determine statistical significance, with a threshold of p < 0.05.

## Western blot

Total protein extracts (20  µg per sample) were separated by SDS-PAGE using 4–12 % gradient polyacrylamide precast gels (Invitrogen, Cergy-Pontoise, France) and transferred onto nitrocellulose membranes. Membranes were blocked for 2 h at room temperature in PBS containing 5 % BSA, then incubated overnight at 4 °C with primary antibodies. Primary antibodies against Cyp27A1, aromatase, and β-actin were obtained from Santa Cruz Biotechnology (CA, USA). After washing, membranes were incubated for 1 h with the appropriate horseradish peroxidase (HRP)-conjugated secondary antibodies, either anti-rabbit or anti-mouse (Santa Cruz Biotechnology, CA, USA).

Protein bands were visualized using enhanced chemiluminescence (Amersham™ ECL Select™, Cytiva Life Sciences) and detected with a CCD camera system (LAS 3000, Fujifilm). Band intensities were quantified using ImageJ software.

All data were expressed as mean ± standard deviation (SD) and analyzed using SigmaPlot software (Systat Software Inc., San Jose, CA, USA). Homogeneity of variance was assessed using the Shapiro–Wilk test. If the assumption of equal variance was met, parametric tests were applied; otherwise, the Mann–Whitney test was used. Statistical significance was defined as p < 0.05.

## Results

### Body and relative reproductive organ weights

No significant difference was observed in F0 generation for body and relative reproductive organ weights. In the F1 generation, body weight was significantly increased (+13.5 %, p ≤ 0.05) in NU groups compared to the control group, whereas the relative weights of reproductive organs (testis, epididymis, seminal vesicle) were significantly decreased (−5.26 %, −15.38 %, and −15.56 %, respectively). Statistical analysis showed a significant decrease in body weight (−10.71 %) and a significant increase in relative testis weight (+15.15 %) in the last generation studied (F2) (Table 1).

**Table 1:** Body weight, relative reproductive organ weights of adult male rats from multigenerational exposure. Results are expressed as mean value ± SD (n = 12–20, depending on the number of live animals). Results are significantly different from the CONTROL group in each generation for: * p ≤ 0.05; **p ≤ 0.005 and ***p ≤ 0.001.

	**F0 generation**		**F1 generation**		**F2 generation**	
	**CONTROL**	**NU**	**CONTROL**	**NU**	**CONTROL**	**NU**
**Body Weight (BW) (g)**	626 ± 83,2 (20)	618 ± 90,3(20)	524 ± 78,6 (20)	595 ± 71,4 * (20)	579 ± 68,9 (19)	517 ± 57,1 ** (20)
**Relative testis weight** (g/100 BW)	0,33 ± 0,03 (17)	0,32 ± 0,05(14)	0,38 ± 0,10 (19)	0,36 ± 0,05 * (14)	0,33 ± 0,05 (17)	0,38 ± 0,06 * (16)
**Relative epididymal weight** (g/100 BW)	0,12 ± 0,01 (17)	0,12 ± 0,0(15)	0,13 ± 0,03 (20)	0,11 ± 0,03 * (19)	0,12 ± 0,02 (19)	0,12 ± 0,03(19)
**Relative seminal vesicle weight** (g/100 BW)	0,34 ± 0,05 (13)	0,38 ± 0,06(12)	0,45 ± 0,11 (14)	0,38 ± 0,08 * (17)	0,33 ± 0,06 (19)	0,33 ± 0,06(18)

## Testicular histology and functional markers

### Testicular histomorphology

**Fig. 2:**
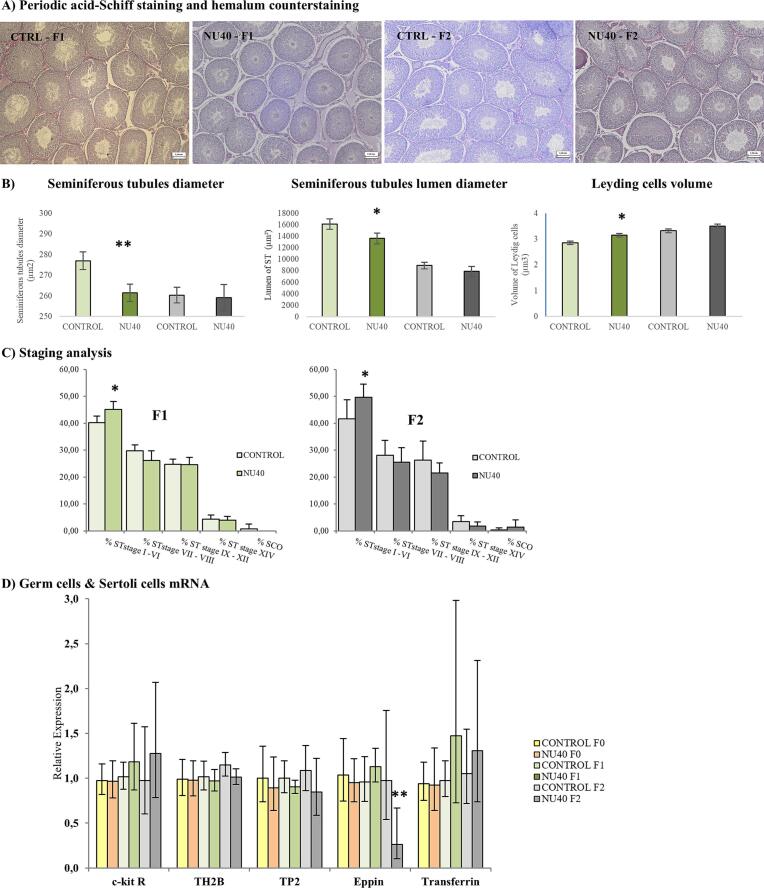
A. Testicular histological section and histomorphometric analysis, including measurements of seminiferous tubule (ST) diameter, ST lumen diameter, and Leydig cell volume, B. Staging analysis of the seminiferous epithelium cycle. Results are expressed as mean ± S.D. (n = 5 to 7 per group), C. Relative mRNA expression levels of germ cell markers (TH2B, TP2, c-kit receptor) and Sertoli cell markers (eppin, transferrin). RT-qPCR results are expressed as mean ± S.D. (n = 10 per group). Statistical significance is indicated as follows: *p ≤ 0.05; **p ≤ 0.005; ***p ≤ 0.001. Color coding: yellow/orange for F0 generation, light/dark green for F1 generation, and light/dark grey for F2 generation.

Histomorphometry analysis showed a statistically significant decrease in the diameter of ST (−5.59 %, p = 0.004) and the lumen of ST (− 15,55 %, p = 0.03) in NU-exposed F1 generations. No difference was observed in the diameter of ST and the lumen of ST in the NU-exposed group of F2 generation. The volume of Leydig cells increased only in the NU-groups from the F1 generation (+10 %, p < 0.001) (Fig. 2A). The staging analysis showed a significant difference between the NU-groups and CONTROL-groups in the percentage of ST in stage I-VI. Specifically, the percentage of ST increased to 45.20 ± 2.95 % *vs* 40.20 ± 2.49 % (p = 0.0197) and 49.67 ± 4.93 % *vs* 41.67 ± 7.03 % (p = 0,046) in the F1 and F2 generation, respectively. No effect was observed for the other stages of the seminiferous cycle (Fig. 2B). No difference was observed in other anomalies (SCO, MGC, vacuoles, SC nuclei) in the F1 and F2 generations.

## Germ and Sertoli cells mRNA expression

RT-qPCR analysis was performed on specific markers of germ cells and Sertoli cells and Sertoli cells secretory products. A statistically significant downregulation of *Eppin* mRNA levels (FC = 0.27; p = 0.005) was observed exclusively in the F2 generation of the exposed group. This gene is associated with Sertoli and spermatogenic cells (Fig. 2D).

## Oxidative stress, Inflammation, BTB Regulators and mortality

**Fig. 3:**
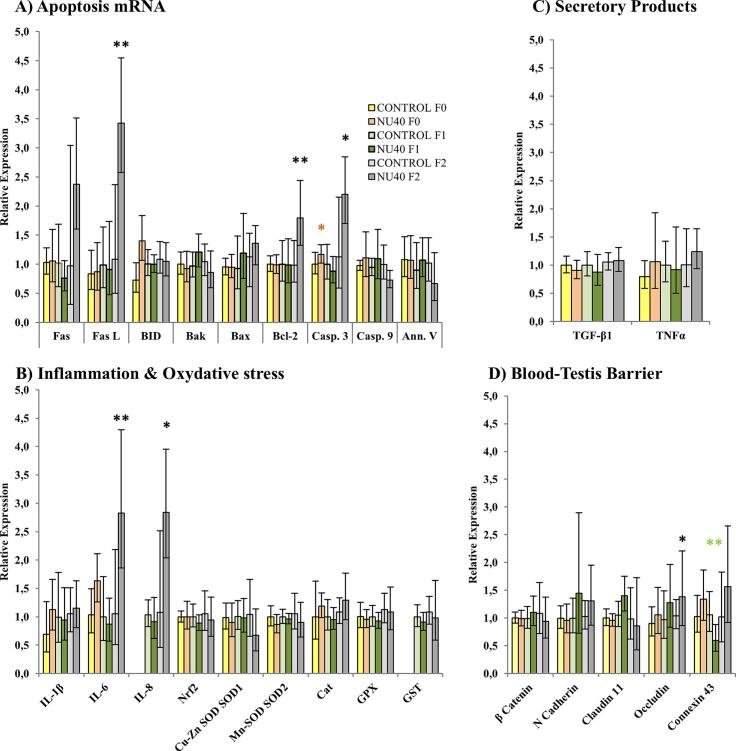
A. Relative mRNA expression levels of apoptosis-related genes (Fas, FasL, BID, Bak, Bax, Bcl-2, caspase-3, caspase-9, annexin V), B. Relative mRNA expression levels of inflammation markers (IL-1β, IL-6, IL-8) and oxidative stress-related genes (Catalase, Cu-Zn SOD [SOD1], Mn-SOD [SOD2], GPX, GST, Nrf2), C. Relative mRNA expression levels of Sertoli cell secretory products (TGF-β1, TNF-α), D. Relative mRNA expression levels of blood-testis barrier components (β-catenin, N-cadherin, claudin-11, occludin, connexin). RT-qPCR results are presented as mean ± S.D. (n = 10 per group). Statistical significance is indicated as follows: *p ≤ 0.05; **p ≤ 0.005; ***p ≤ 0.001. Color coding: yellow/orange for F0 generation, light/dark green for F1 generation, and light/dark grey for F2 generation.

Among the apoptotic antigen family members, *FasL* was significantly upregulated in the F2 generation (FC = 3.15, p = 0.007). Similarly, *caspase 3* expression increased in both the F0 and F2 generations (FC = 1.17 and 1.95, p = 0.048 and 0.023, respectively). The intrinsic apoptotic pathway, which can be triggered by pore-forming pro-apoptotic proteins such as Bcl-2, was also affected, showing an increased mRNA expression in the F2 generation (FC = 1.82, p = 0.002) (Fig. 3A).

With regard to inflammatory and oxidative stress markers secreted by Sertoli cells, only the pro-inflammatory cytokines *IL-6 and IL-8* showed a strong and statistically significant induction in the F2 generation (FC = 2.69 and 2.64, p = 0.003 and 0.015, respectively) (Fig. 3, Fig. 3).

Finally, analysis of mRNA levels for β-catenin, N-cadherin and claudin-11, markers of the blood–testis barrier (BTB) junctions, revealed no major deregulation, except for connexin 43, which was downregulated in the F1 generation, followed by an upregulation of occludin, associated with tight junctions, in the F2 generation (FC = 0.53 and 1.33; p = 0.003 and 0.024, respectively) (Fig. 3 **D**).

## Hormonal effects

### Sex hormone levels and steroidogenesis

Sexual hormones were also assayed in plasma and testicular extracts in each generation. Testosterone levels were modified in only one generation (F1) and decreased significantly in plasma (–32 %, p < 0.05) and in the testis (−41 %, p < 0.05). Significant increases were also detected for estradiol concentrations in two generations: in plasma and intratesticular compartments in the F0 generation (+27 % and + 36 %, respectively) and in the intratesticular compartment in the F2 generation (+55 %). Gonadotropin levels were modified only in the last generation (F2) in male rats exposed to NU in comparison with CONTROL-F2 groups: LH concentration decreased significantly (−58 %, p = 0.001) and FSH levels increased significantly (+49 %, p = 0.001) (Table 2).

**Table 2:** Hormonal level of adult male rats from multigenerational exposure. Results are expressed as mean value ± SD (n = 7–20, depending on the number of samples available). Results are significantly different from the CONTROL group in each generation for: * p ≤ 0.05; **p ≤ 0.005 and ***p ≤ 0.001.

	**F0 generation**		**F1 generation**		**F2 generation**	
	**CONTROL**	**NU**	**CONTROL**	**NU**	**CONTROL**	**NU**
**LH** (ng.mL^−1^)	0.450 ± 0.33 (20)	0.653 ± 0.40 (19)	0.434 ± 0.15 (19)	0.425 ± 0.24 (20)	0.683 ± 0.62 (17)	0.288 ± 0.18 *** (12)
**FSH** (pg.mL^−1^)	6.53 ± 1.81 (15)	7.67 ± 1.77* (15)	11.00 ± 2.08 (19)	11.59 ± 1.75 (20)	7.26 ± 1.41 (10)	10.82 ± 1.94*** (10)
**Testosterone**						
Plasma level (ng.mL^−1^)	0.969 ± 0.60 (18)	1.099 ± 0.59 (14)	1.779 ± 0.66 (10)	1.213 ± 0.40* (18)	1.088 ± 0.42 (12)	1.184 ± 0.37 (15)
Intratesticular level (ng.g^−1^)	0.207 ± 0.13 (10)	0.242 ± 0.14 (10)	0.227 ± 0.1 (10)	0.133 ± 0.04* (9)	0.219 ± 0.13 (10)	0.272 ± 0.19 (10)
**Estradiol**						
Plasma level (pg.mL^−1^)	54.34 ± 18.37 (17)	68.92 ± 13.62* (17)	22.84 ± 6.35 (9)	23.99 ± 4.4 (12)	48.55 ± 8.52 (10)	53.11 ± 8.99 (10)
Intratesticular level (pg.g^−1^)	0.179 ± 0.05 (9)	0.245 ± 0.04* (8)	0.281 ± 0.06 (10)	0.266 ± 0.02 (9)	0.255 ± 0.09(7)	**0.395 ± 0.08** ** (8)

**Fig. 4:**
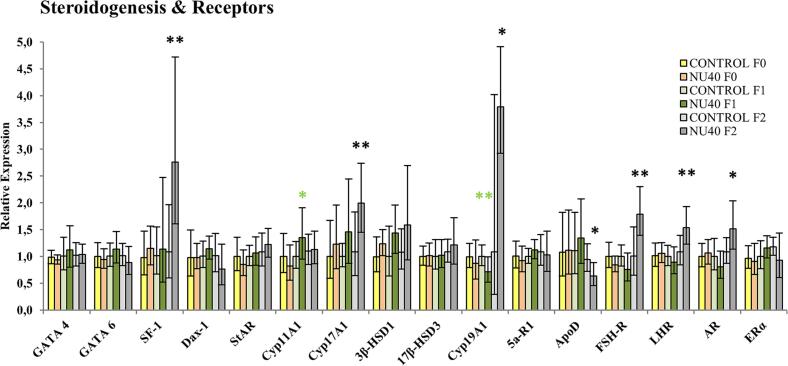
Relative mRNA expression levels of genes involved in steroid hormone metabolism (GATA4, GATA6, SF-1, Dax-1, StAR, Cyp11A1, Cyp17A1, 3β-HSD1, 17β-HSD3, Cyp19A1, 5α-R1, ApoD) and nuclear receptors (FSH-R, LRH, AR, ERα). RT-qPCR results are presented as mean ± S.D. (n = 10 per group). Statistical significance is indicated as follows: *p ≤ 0.05; **p ≤ 0.005; ***p ≤ 0.001. Color coding: yellow/orange for F0 generation, light/dark green for F1 generation, and light/dark grey for F2 generation.

RT-qPCR analysis revealed a significant upregulation of several key genes involved in the early stages of steroidogenesis in the F2 generation, including *SF-1* (FC = 2.54, p = 0.004) and *Cyp17A1* (FC = 1.84, p = 0.012). Additional genes such as *FSHR*, *LHR*, and *AR* also showed increased expression (FC = 1.78, 1.42, and 1.40; p = 0.003, 0.008, and 0.02, respectively). In contrast, *ApoD* expression was significantly downregulated (FC = 0.67, p = 0.016). Regarding aromatase (*Cyp19A1*), its mRNA levels were markedly elevated in the F2 generation (FC = 3.5, p = 0.05; Fig. 4), although no corresponding changes were detected at the protein level by Western blot or ELISA (n = 9–10 per group; data not shown). In the F1 generation, only *Cyp11A1* and *Cyp19A1* showed significant dysregulation, with fold changes of 1.35 and 0.72, respectively (p = 0.041 and 0.014).

## Vitamin D metabolism

**Fig. 5:**
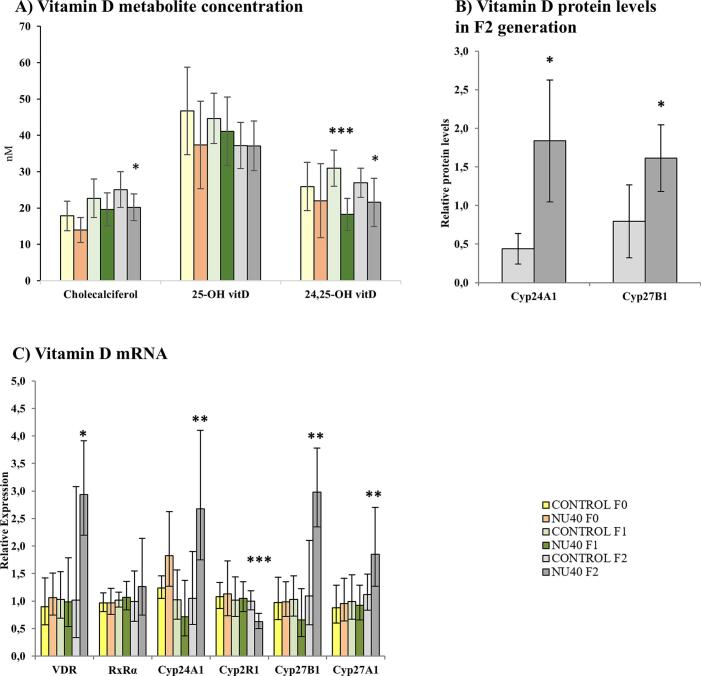
A. Relative mRNA expression levels of genes involved in Vitamin D_3_ metabolism (VDR, RxRα, Cyp24A1, Cyp2R1, Cyp27B1) and cholesterol metabolism (Cyp27A1). RT-qPCR results are presented as mean ± S.D. (n = 10 per group), B. Relative protein levels of key enzymes involved in Vitamin D3 metabolism, C. Plasma concentrations (nmol/L) of Vitamin D metabolites (cholecalciferol, 24,25-dihydroxyvitamin D_3_, 25-hydroxyvitamin D_3_). All results are expressed as mean ± S.D. (n = 10 per group). Statistical significance is indicated as follows: *p ≤ 0.05; **p ≤ 0.005; ***p ≤ 0.001. Color coding: yellow/orange for F0 generation, light/dark green for F1 generation, and light/dark grey for F2 generation.

No changes in plasma levels of cholecalciferol, 24,25(OH) _2_D_3_, or 25(OH)D_3_, the major circulating form of vitamin D, were observed in the F0 generation. However, in the F1 generation, plasma levels of 24,25(OH) _2_D_3_ were significantly reduced by 41 %, and by 20 % in the F2 generation, compared to the control groups. Additionally, cholecalciferol levels were significantly decreased by 19 % in the F2 generation only (Fig. 5 **A**). In the F2 generation, protein levels of key enzymes involved in vitamin D metabolism were significantly elevated in the testes, including CYP27B1 (FC = 2.03, p = 0.032; n = 5 per group) and CYP24A1 (FC = 4.20, p = 0.041; n = 3 per group), while CYP27A1 protein levels remained unchanged (Fig. 5 **B**). Similarly, mRNA expression levels were exclusively altered in the F2 generation. Specifically, the mRNA levels of the vitamin D receptor (*VDR*), *CYP24A1*, and *CYP27B1* were significantly upregulated (FC = 2.89, 2.6, and 2.7; p = 0.018, 0.005, and 0.003, respectively). Notably, *Cyp27A1* mRNA was also significantly overexpressed (FC = 1.7, p = 0.01), consistent with the observed increase in protein levels. In contrast, *Cyp2R1* mRNA, involved in the conversion of vitamin D3 (cholecalciferol) to 25-hydroxyvitamin D3 (25(OH)D3), was significantly downregulated in the F2 generation (Fig. 5 **C**).

## Discussion

According to the World Health Organization (WHO), environmental degradation poses risks that include reduced fertility in directly exposed populations and their descendants (World Health Organization, 2023). This study was designed to simulate chronic environmental exposure to uranium, similar to conditions experienced by populations living in contaminated areas, such as post-military conflict zones or regions affected by mining activities. In our model, rats were exposed to uranium through drinking water at a concentration of 40 mg/L. While this level is higher than the estimated daily intake for humans in uncontaminated areas (1–4 µg from food and water), it remains relevant when considering highly contaminated environments, such as the reported Finnish well where uranium concentrations reached 20 mg/L. Furthermore, given that rodents exhibit an intestinal absorption rate approximately ten times greater than humans, the experimental concentration employed in this study can be considered physiologically meaningful for modeling chronic exposure scenarios.

This study suggests that multigenerational exposure to natural uranium may impair male reproductive function in rats, potentially through hormone-disrupting mechanisms. This hypothesis has previously been proposed in the context of chronic exposure to depleted uranium (Legendre et al., 2016). Recent investigations using spermatozoa from the same cohort of animals as in the present study have revealed morphometric abnormalities and metabolomic biomarkers associated with uranium exposure across all examined generations (F0, F1, and F2) (Legendre et al., 2019, Grison et al., 2022). Additionally, a significant reduction in gestation rates was observed in the F1 generation (Legendre et al., 2019). These findings underscore the need for further research into testicular steroidogenesis and spermatogenesis, to identify specific markers of male fertility that may be affected.

In the present study, disruptions in testicular function were observed, offering valuable insights into the reprotoxic effects of uranium. First, numerous histological and weight-related effects were observed. Notably, a reduction in seminiferous tubule (ST) diameter and lumen size was detected exclusively in the F1 generation, which may be associated with the observed decrease in relative testicular weight. During puberty, the expansion of testicular volume and ST diameter is typically driven by germ cell proliferation and the onset of spermatogenesis, processes regulated by the combined action of follicle-stimulating hormone (FSH) and testosterone. (Li et al., 2024). Regarding tissue effects in the F1 generation, histological findings suggest that prenatal exposure to uranium may have interfered with the normal process of testicular maturation. (Wu et al., 2025).

Compared to the F1 generation, the effect was reversed in the F2 generation and was observed only in the testis, rather than in the two other reproductive organs, the epididymis and the seminal vesicle. Specifically, an increase in relative testicular weight was noted. This change could stem from either a mechanical effect, such as obstruction of the vas deferens, or a physiological effect, like impaired water resorption. Endocrine disruptors, such as atrazine or betamethasone, are known to exert estrogenic or anti-estrogenic effects, which can influence fluid resorption and contribute to such alterations in testicular morphology (Hess et al., 1997). In our study, intratesticular levels of estrogens, particularly estradiol, were significantly elevated in the F2 generation. Estradiol is synthesized in Leydig cells via the aromatization of testosterone, a reaction catalyzed by the enzyme aromatase (CYP19A1). In this experimental study, we observed a consistent upregulation of cyp19a1 mRNA expression, suggesting that Leydig cells may be hyperstimulated and rapidly convert testosterone into estradiol. Elevated estradiol levels could contribute to altered fluid resorption, potentially explaining the observed increase in testicular weight and highlighting a possible endocrine disruption mechanism associated with uranium exposure. However, protein levels remained unchanged, and enzyme activity was not assessed, which limits confirmation of this hypothesis.

Other histological effects were observed in the seminiferous cycle of both uranium-exposed F1 and F2 generations. Specifically, the percentage of ST in stage I-VI was higher in these two generations, which could be linked to the slowdown of the cycle due to the blockage at the beginning of its process. The transition from stage VI to stage VII of spermatogenesis is regulated by intratesticular levels of testosterone and follicle-stimulating hormone. Kerr et al. demonstrated that in hypophysectomized rats, testis weight declined by 50 %, spermatogenesis was severely disrupted, and only 18 % of the tubules contained spermatids, which were confined to stages I–VI of the spermatogenic cycle (Kerr, 1992, Kerr et al., 1992). Kerr et al. also showed that testosterone and FSH exert independent, synergistic and stage-dependent effects on spermatogenesis in the rat testis. In our model, the high percentage of stages I-VI in ST could be related to the decreased testosterone levels observed in the F1 generation and the modified FSH and LH levels shown in the F2 generation. Similar sensitivity of the hypothalamic-pituitary–testicular (HPT) axis was previously demonstrated in male rats, but only after lifelong exposure to a high dose of uranium (120 mg/L) in an adult exposure model (Legendre et al., 2016). In our multigenerational model of exposure, we have shown for the first time the disruption of the HPT axis in the third generation (F2). Indeed, in the F2 generation, the gene expression of steroidogenesis markers (*sf-1, cyp17a1, 3β-hsd1, cyp19a1, FSHR, LHR* and *AR*) was upregulated, while markers of germ and Sertoli cells (eppin) were downregulated. FSH is required to provide an adequate number of primary spermatocytes capable of maturing into round spermatids, which can then proceed through spermiogenesis if testosterone is available. FSH increases the sensitivity (presumably of the Sertoli cells) to testosterone, for example, by increasing the number of androgen receptors and enhancing responsiveness to testosterone activation (Verhoeven and Cailleau, 1988). Together, these modifications at various levels of gene expression and hormone regulation suggest a potential regulatory mechanism involving the spermatogenesis and steroidogenesis in the F2 rats of our multigenerational exposure model.

In addition to testicular alterations, other key markers involved in the protection and regulation of testicular homeostasis, such as oxidative stress, inflammation, cell mortality, and blood–testis barrier (BTB) integrity, were predominantly affected in the F2 generation. These findings support the hypothesis of multigenerational dysregulation at the gene expression level, involving several factors critical to reproductive function. The observed changes are consistent with the long-term consequences of chronic uranium exposure initiated in the F0 generation.

Uranium’s disruptive effect on vitamin D metabolism has been demonstrated (Tissandie et al., 2007), and the role of vitamin D in reproductive function is well established (Yahyavi et al., 2024, Blomberg, 2014). It is involved in estradiol biosynthesis, luteinizing hormone (LH) responsiveness (Hofer et al., 2014, Kinuta et al., 2000); pituitary function, aromatase expression (Krishnan et al., 2010), and sperm motility (Blomberg, 2014). Building on prior findings, our model revealed dysregulation of vitamin D precursors (cholecalciferol, 24,25-dihydroxyvitamin D_3_) as well as altered expression of key testicular genes and proteins (*Cyp24a1, Cyp27b1, Cyp27a1, VDR*) in the F2 generation. This is the first report of such molecular changes in testicular tissue under multigenerational uranium exposure, emphasizing the need for further investigation into the genomic and hormonal roles of vitamin D in male reproductive health (Zanatta et al., 2011).

Given the established sensitivity of vitamin D metabolism to uranium exposure and its pivotal role in male reproductive physiology, we hypothesize that vitamin D may act as a key mediator of the observed reproductive disruptions. Accordingly, further investigation into the role of vitamin D in testicular function is warranted. In particular, in vitro models using Leydig, Sertoli, or germ cell cultures could be instrumental in elucidating the direct effects of uranium on steroidogenesis, oxidative stress, and vitamin D signaling pathways. Such experimental approaches would also facilitate the identification of causal mechanisms by validating the molecular alterations observed *in vivo*.

A decrease in seminal vesicle weight, an androgen-dependent and endocrine-sensitive organ (Reproductive and Toxicology, 1998); was observed exclusively in the F1 generation, further supporting uranium’s endocrine-disrupting potential. Epididymal weight also declined in F1, although it was not further analyzed. Given previous findings of altered sperm metabolome and DNA methylation in the F2 generation (Legendre et al., 2019, Grison et al., 2022); additional studies focusing on epididymal function and sperm motility are warranted.

Although this study focused primarily on the male reproductive system, it is important to acknowledge that infertility originates from both male and female factors. Our model revealed significant epigenetic modifications not only in the testis but also in ovarian tissues across generations (Elmhiri et al., 2018). Furthermore, the reduced pregnancy rates observed in F1 (Legendre et al., 2019) and the disruption of estrous cycles, characterized by a 58 % decrease in estrus (p = 0.007) and a 127 % increase in diestrus (p = 0.016), underscore the need for deeper investigation into female reproductive health under chronic uranium exposure. These findings highlight the importance of expanding future research to comprehensively address the impact of uranium on female gonadal function and overall reproductive outcomes (see Supplemental Fig. 1).

## Conclusion

For the first time, this study showed the multigenerational effects of chronic exposure to non-nephrotoxic doses of uranium on male reproductive function using a preclinical rat model. Our findings reveal that uranium exposure disrupts spermatogenesis, with the most pronounced alterations observed in the F2 generation. This generation was exposed through both parental and grandparental lineages during critical developmental windows, including the fetal and neonatal periods, or throughout the lifespan, respectively. In F2 offspring, several key indicators of reproductive function were affected, including steroidogenesis and testicular homeostasis.

Finally, the more pronounced effects observed in the F1 and F2 generations, compared to the chronically exposed F0 generation, underscore the potential for long-term and intergenerational risks associated with environmental contaminants. These findings highlight the importance of continued research using diverse experimental models and complementary epidemiological studies to better understand the underlying mechanisms of toxicity. Strengthening environmental and public health policies, including radiation protection strategies, is essential to mitigate reproductive risks linked to chronic uranium exposure and similar pollutants.

## CRediT authorship contribution statement

**Audrey Legendre:** Conceptualization, Methodology, Validation, Formal analysis, Investigation, Data curation, Writing – original draft, Writing – review & editing, Visualization, Supervision. **Céline Gloaguen:** Methodology, Data curation, Writing – original draft, Writing – review & editing, Visualization. **Dimitri Kereselidze:** Methodology, Formal analysis, Writing – review & editing. **Nawel Saci:** Methodology, Formal analysis, Writing – review & editing. **Sophia Murat El Houdigui:** Methodology, Formal analysis, Writing – review & editing. **Pascal Froment:** Methodology, Formal analysis, Writing – review & editing. **Christelle Elie:** Methodology, Formal analysis, Writing – review & editing. **Catherine Defoort:** Methodology, Formal analysis, Writing – review & editing. **Philippe Lestaevel:** Methodology, Formal analysis, Writing – review & editing. **Mohamed Amine Benadjaoud:** Methodology, Formal analysis, Writing – review & editing. **Maâmar Souidi:** Conceptualization, Methodology, Validation, Formal analysis, Data curation, Writing – original draft, Writing – review & editing, Visualization, Supervision. **Stéphane Grison:** Conceptualization, Methodology, Validation, Formal analysis, Data curation, Writing – original draft, Writing – review & editing, Visualization, Supervision, Project administration.

## Funding

This research received no external funding.

## Declaration of competing interest

The authors declare that they have no known competing financial interests or personal relationships that could have appeared to influence the work reported in this paper.
