# Supplementary material for: Multigenerational effects of uranium exposure reveal stronger testicular dysregulation in the second generation
Source: Curr Res Toxicol. 2025 Dec 26;10:100279. doi: 10.1016/j.crtox.2025.100279 (PMC12856330; doi:10.1016/j.crtox.2025.100279)
Supplement: Supplementary Data 1 [file mmc1.docx]

*Supplemental Method, TABLE 1: Sequence of primer sets for gene expression studies in the testis.*

| **Function** | **Gene (accession no.)** | **Forward** | **Reverse** | **Amplicon size (bq)** | **Reference** |
| --- | --- | --- | --- | --- | --- |
| Apoptosis / Pro-Apoptosis | Bid (NM001196) | GAGCTCGGGTCCAAGTGTC | CTCAGAGTCCATGACGCAGG | 113 | Primer-BLAST |
| Apoptosis / Pro-Apoptosis | Caspase 3 (XM_006253130.2) | AAATTCAAGGGACGGGTCAT | ATTGACACAATACACGGGATCTGT | 183 | Primer-BLAST |
| Apoptosis | Caspase 9 (NM_031632.1) | ACAAGGCCTTCGACAGTG | GTACCAGGAACCGCTCTT | 176 | Primer-BLAST |
| Apoptosis (precosis) | Annexin V (AB001349) | TCAGAGTAGCAGCTCCCTTGTTTG | CTCCAAATCCTGAAACGGGAATGC | 106 | Primer-BLAST |
| Apoptosis / Anti-apoptosis / GC (SPC, SPT) products | BCl2 (NM_021850.2) | GGCCTTTAGCAAACAGGAGCA | AGCTTCTGGGAATCCAGACCAA | 136 | Song et al., 2011 |
| Apoptosis / Pro-Apoptosis | BAK (NM_053812.1) | GACGATATTAATCGGCGCTACGAC | GCTGGTAGACATACAGGGCCAGA | 187 | Song et al., 2011 |
| Apoptosis / Pro-Apoptosis | BAX (NM_017059.2) | AGACACCTGAGCTGACCTTGGAG | GTTGAAGTTGCCATCAGCAAACA | 197 | Song et al., 2011 |
| Apoptosis / Pro-Apoptosis / SC products | FAS (NM_139194.2) | TTCTGAGCAGTTGTTGTCGGTTT | CGAATGCAAGGGACTGATAGC | 81 | Primer-BLAST |
| Apoptosis / Pro-Apoptosis / SC products | FASL (XM_008769607.1) | TTGCAAGACTGACCCCGG | AAGGCGGCCTTGTGATCA | 76 | Primer-BLAST |
| BTB / Sertoli cells | Transferrin (NM_001013110) | GCTGTGGCCAGTTTCTTCTC | CCACATCTCCACCTCCATCT | 163 | Olumide and Raji,2011 |
| BTB / Sertoli cells : AJs | N-cadherin (XM_006254441.1) | GCGGCCTTGCTTCAGGCATC | CTGGCCTTCGTGCACGTCCT | 114 | Nascimento et al., 2012 |
| BTB / Sertoli cells : AJs | Beta-catenin (NM_053357.2) | TGGACAATGGCTACTCAAGCTGAC | GGGATGAGCAGCGTCAAACTGCGT | 363 | Lee et al., 2003 |
| BTB / Sertoli cells : Gap Js | Connexin43 (XM_006256503.2) | TGGCTGCTCCTCACCAACGGC | GGTCATCAGGCCGAGGCCTGC | 332 | Xia et al., 2009 |
| BTB / Sertoli cells : Gap Js | Connexin43 (XM_006256503.2) | TAAAAAGTTCCGCTGCTCGT | AGCAGGCTAGGCAAAGTTGA | 186 | [AH003191.2](http://www.ncbi.nlm.nih.gov/nucleotide/338825643?report=genbank&log$=nucltop&blast_rank=1&RID=RMS0Z237015) |
| BTB / Sertoli cells : TJs | Occludin (NM_031329.2) | GCTCAGGGAATATCCACCTATCA | CACAAAGTTTTAACTTCCCAGACG | 442 | Gye and Ohsako,2003 |
| BTB / Sertoli cells : TJs | Claudin11 (NM_053457.2) | GATTGGCATCATCGTCACAACG | AGCCAGCAGAATAAGGAGCAAC | 340 | Florin et al., 2005 |
| Germ cells and Sertoli cells | Eppin (NM_001109457.1) | GAAGTTTTCCAGATTGTGAGC | CCGTTCAGGTGGAATTGCTT | 407 | Silva, Patrão et al. 2012 |
| Germ cells : Pa | TH2B (NM_022643.1) | TGAGACGTTGGAGTGGACAA | GTAACTCTCTTCGCGGCATC | 152 | Marret et al., 1998 |
| Germ cells : Spd r | TP2 (NM_017057.2) | CATTCCCCTAGTGATGGCTATCTCC | AGGAAAGGTGAGCAAGAGAAAGGCG | 152 | Legendre et al., 2010 |
| Germ cells : Spg A (B, Int) | c-kitR (NM_022264.1) | GCATCACCATCAAAAACGTG | GATAGTCAGCGTCTCCTGGC | 332 | Dym et al., 1995 |
| Inflammation and Oxydative Stress | Nrf2 (AF037350) | TGGGTTCAGTGACTCGGAAAT | TGGCTGTGCTTTAGGTCCATT | 78 | Poisson et al., 2014 |
| Inflammation and Oxydative Stress | CAT (NM_012520.2) | GAGAGGAAACGCCTGTGTGAG | AAGAGCCTGGACTCGGGCCC | 126 | Poisson et al., 2013 |
| Inflammation and Oxydative Stress | SOD1 (NM_017050.1) | GATTAACTGAAGGCGAGCAT | CCGCCATGTTTCTTAGAGT | 109 | Poisson et al., 2013 |
| Inflammation and Oxydative Stress | SOD2 (NM_017051.2) | ACGCGACCTACGTGAACAATCT | CAGTGCAGGCTGAAGAGCAA | 101 | Poisson et al., 2013 |
| Inflammation and Oxydative Stress | GPx1 (Se-dependent Gpx) (NM_030826.3) | TGCAATCAGTTCGGACATCA | ACCATTCACCTCGCACTTC | 126 | Itoh et al., 2004 |
| Inflammation and Oxydative Stress | GST (NM012577) | TGG AGA CCT CAC CCT TTA CCA A | CCA CCC CAT CAT TCA CCA TA | 119 | Poissonet al., 2013 |
| Inflammation and Oxydative Stress | IL-1beta (NM_031513) | CACACTAGCAGGTCGTCATCATC | ATGAGAGCATCCAGCTTCAAATC | 214 | Fu et al. 2014 |
| Inflammation and Oxydative Stress | IL-6 (NM_012589) | ACAAGTCGGAGGCTTAATTACACAT | TTGCCATTGCACAACTCTTTTC | 72 | Lestaevel et al. 2008 |
| Inflammation and Oxydative Stress / pro-inflammation | IL-8 (NM_001394590.1) | GACTGTTGTGGCCCGTGAG | CCGTCAAGCTCTGGATGTTCT | 83 | Dublineau et al. 2006 |
| Inflammation/BTB regulation | TGFβ1 (NM_021578.2) | CAGGTGTTGAGCCCTTTCCA | TCCCAAACGTCGAGGTGAC | 94 | Primer-BLAST |
| Inflammation/BTB regulation | TNFalpha (NM_011345) | CAGAGCAATGACTCCAAAGTA | CAAGAGCCCTTGCCCTAA | 181 | Fu et al. 2014 |
| Reference | Beta-actin (NM_031144.3) | TTCAACACCCCAGCCATGT | TGGTACGACCAGAGGCATACAG | 67 | Primer-BLAST |
| Reference | Beta 2-microglobulin (NM_012512.2) | ACATCCTGGCTCACACTGAA | ATGTCTCGGTCCCAGGTG | 109 | Primer-BLAST |
| Reference | HPRT (NM_012583) | GCT CGA GAT GTC ATG AAG GAG A | TCA GCG CTT TAA TGT AAT CCA GC | 148 | Primer-BLAST |
| Steroid Hormone Receptor | Erα (NM_012689.1) | TCCGGCACATGAGTAACAAA | TGAAGACGATGAGCATCCA | 109 | Quignot et al., 2012 |
| Steroid Hormone Receptor | LHR (NM_012978.1) | TTATTCCGCCATCTTTGAGG | ACAGGGGTTGAAAGCATCTG | 112 | Quignot et al., 2012 |
| Steroid Hormone Receptor | FSHR (NM_199237.1) | CTCATCAAGCGACACCAAGA | ACCTTGAGGGAGGCAGAAAT | 108 | Quignot et al., 2012 |
| Steroid Hormone Receptor | AR (NM012502) | GGGTTGGCGGTCCTTCA | GAAAACCAGGTCAGGTGCAAAG | 64 | Grignard et al., 2008 |
| Steroid Hormone Receptor | ApoD (NM012777) | GATGGCGACCATGCTGTTG | TGTCCTTCGGTTGTGGTGAA | 63 | Primer Express |
| Steroidogenesis | Cyp11a1 (NM017286) | CTCTGCAATGGAACCTTTATGAAAT | CTTCAGCCCGCAGCATCT | 66 | Grignard et al., 2008 |
| Steroidogenesis | Cyp17a1 (NM012753) | TGGCTTTCCTGGTGCACAATC | TGAAAGTTGGTGTTCGGCTGAAG | 90 | Primer-BLAST |
| Steroidogenesis | HSD3β1 (NM001007719) | CCCAGGCAGACCATCCTAGAT | ACGCAGGCCTCCAATATGTTC | 62 | Grignard et al., 2008 |
| Steroidogenesis | HSD17β3 (NM054007) | TCGGGAAAGCCTATTCATTTGA | TCCGGCTGATAAGTACAACATTGA | 63 | Grignard et al., 2008 |
| Steroidogenesis | 5αR1 (NM017070) | TCCTGGTCACCTTTGTCTTGGC | GTTTCCCCTGGTTTTCTCAGATTC | 128 | Grignard et al., 2008 |
| Steroidogenesis | Cyp19a1 (NM017085) | ACCATCATGGTCCCGGAAA | AGGCCCATGATCAGCAGAAG | 62 | Grignard et al., 2008 |
| Steroidogenesis | StaR (NM_031558.3) | TTCTCAACTGGAAGCAACACTCTAC | ACCTGGCACCACCTTACTTAGC | 166 | Zhang et al., 2013 |
| Steroidogenesis | Gata6 (NM_019185.1) | CATGCGGTCTCTACAGTAAGATGAA | ATGAAGGCACGCGCTTCT | 72 | Grignard et al., 2008 |
| Steroidogenesis | Gata4 (NM144730) | GATGGGACAGGACACTACCTATGC | GGCGCTGAGGCTTGATGA | 88 | Primer-BLAST |
| Steroidogenesis | SF-1 (AB009575) | TGCTTACCAGACCTTGGGATGT | GGTGCTCGTGTGGAAATGG | 65 | Primer Express |
| Steroidogenesis | Dax-1 (NM053317) | CCGATGTTGTCACTGAACTCTTTT | CACAGAGCATCTCCAGCATCAT | 81 | Grignard, Gueguen et al. 2008 |
| Vitamin D Hormone | Cyp27B1 (NM_053763.1 ) | CAACTCGGGGGTTAACTAACAG | AAGCATGGAAGGATCAGTGG | 69 | Douard et al 2014 |
| Vitamin D Hormone | Cyp24A1 (NM_201635.3) | TGGATGAGCTGTGCGATGA | TGCTTTCAAAGGACCACTTGTTC | 75 | Anderson et al, 2003 |
| Vitamin D Hormone | Cyp27a1 (NM_178847) | GGAAGGTGCCCCAGAACAA | GCGCAGGGTCTCCTTAATCA | 71 | Tissandie et al. 2007 |
| Vitamin D Receptor | VDR (NM_017058.2) | TGACCCCACCTACGCTGACT | CCTTGGAGAATAGCTCCCTGTACT | 79 | Anderson et al, 2003 |
| Vitamin D Receptor | RXRalpha (NM_012805) | CGCAAAGACCTGACCTACACC | TCCTCCTGCACAGCTTCCC | 133 | Souidi et al. 2005 |
