# Supplementary figures and images for: Multigenerational effects of uranium exposure reveal stronger testicular dysregulation in the second generation
Source: Curr Res Toxicol. 2025 Dec 26;10:100279. doi: 10.1016/j.crtox.2025.100279 (PMC12856330; doi:10.1016/j.crtox.2025.100279)

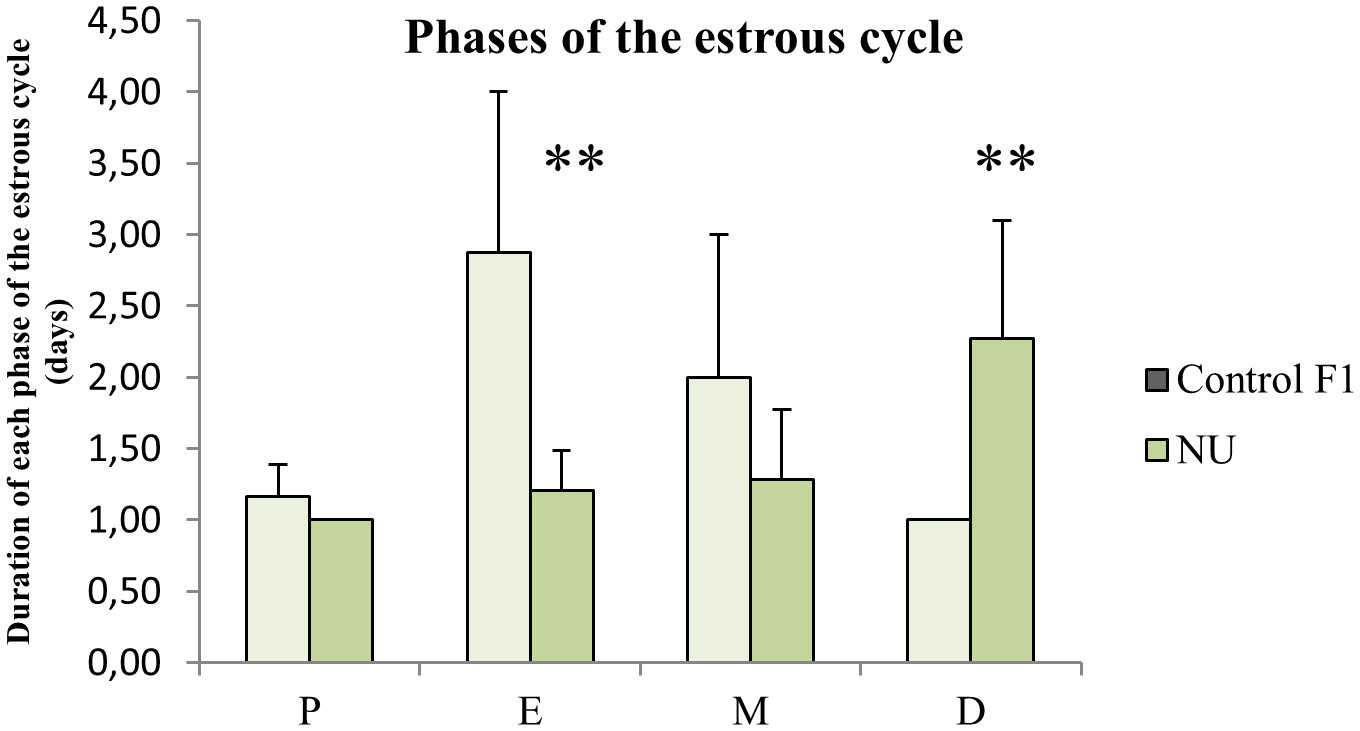

Supplement: Supplementary Figure 2 [file mmc2.jpg]
